# Modelling HIV modes of transmission in Iran

**DOI:** 10.1186/1742-4690-9-S1-P121

**Published:** 2012-05-25

**Authors:** Maryam Nasirian, Ali-Akbar Haghdoost, Fardad Doroudi, Mohammad Mehdi Gooya, Abbas Sedaghat, Eshagh Dortaj Rabbori

**Affiliations:** 1 Kerman University of Medical Science, Kerman, Iran

## Introduction

There is inadequate information on high-risk populations even though prevention programmes are the mainstay of the national response to the HIV epidemic in these populations. We used the mode of transmission (MOT) model to understand the sources of new HIV infections and use this information for programme planning.

## Material and methods

We systematically searched published and grey literature to find the best values for the input parameters required by the MOT model. The data were discussed by a group of national experts before being fed into the MOT model. Using the Monte Carlo technique, we computed the 95-percent uncertainty level (UCL) for the outputs of the MOT.

## Results

The MOT model estimates that 9136 new HIV infections will occur in Iran in 2010 (UCL 6831-11757). Fifty-six percent (UCL 47.7–61.6%) of new infections were among injecting drug users and 12 percent (UCL 9.5–15%) among their sexual partners. The major routes of direct and indirect HIV transmission in Iran are unsafe injection (68%) and sexual contact (34% heterosexual and 10% same-sex). If current coverage for safe injection among IDUs increased from 80 to 95 percent, the number of new HIV infections in this group would decrease by almost 75%.

## Conclusion

IDUs remain the key population at highest risk of HIV infection in Iran, so programme coverage for IDUs and their spouses needs to be increased. And as the sexual transmission of HIV increasingly contributes to the pool of new infections, serious measures are required to reduce sexual transmission of HIV among the relevant key populations.

